# Targeting canine bladder transitional cell carcinoma with a human bladder cancer-specific ligand

**DOI:** 10.1186/1476-4598-10-9

**Published:** 2011-01-27

**Authors:** Tzu-yin Lin, Hongyong Zhang, Sisi Wang, Li Xie, Bin Li, Carlos O Rodriguez, Ralph de Vere White, Chong-xian Pan

**Affiliations:** 1 Division of Hematology and oncology, Department of Internal Medicine, University of California-Davis Cancer Center, Sacramento, CA 95817, USA; 2 Department of Pharmacology, Norman Bethune College of Medicine, Jilin University, Changchun, PR China; 3 Health-coming Co. Ltd, Unit 3, No. 128 Shuang Lian Road, Haining City, Zhejiang 314400, PR China; 4 Department of Surgical and Radiological Sciences, School of Veterinary Medicine, University of California-Davis, Davis, CA 95616, USA; 5 Department of Urology, University of California-Davis Cancer Center, Sacramento, CA 95817, USA; 6 VA Northern California Health Care System, 10535 Hospital Way, Mather, CA 95655, USA

## Abstract

**Objective:**

To determine if a human bladder cancer-specific peptide named PLZ4 can target canine bladder cancer cells.

**Experimental Design:**

The binding of PLZ4 to five established canine invasive transitional cell carcinoma (TCC) cell lines and to normal canine bladder urothelial cells was determined using the whole cell binding assay and an affinitofluorescence assay. The WST-8 assay was performed to determine whether PLZ4 affected cell viability. *In vivo *tumor-specific homing/targeting property and biodistribution of PLZ4 was performed in a mouse xenograft model via tail vein injection and was confirmed with *ex vivo *imaging.

**Results:**

PLZ4 exhibited high affinity and specific dose-dependent binding to canine bladder TCC cell lines, but not to normal canine urothelial cells. No significant changes in cell viability or proliferation were observed upon incubation with PLZ4. The *in vivo *and *ex vivo *optical imaging study showed that, when linked with the near-infrared fluorescent dye Cy5.5, PLZ4 substantially accumulated at the canine bladder cancer foci in the mouse xenograft model as compared to the control.

**Conclusions and Clinical Relevance:**

PLZ4 can specifically bind to canine bladder cancer cells. This suggests that the preclinical studies of PLZ4 as a potential diagnostic and therapeutic agent can be performed in dogs with naturally occurring bladder cancer, and that PLZ4 can possibly be developed in the management of canine bladder cancer.

## Background

Recently, we have developed a bladder cancer-specific peptide named PLZ4 (the amino acid sequence: cQDGRMGFc) that specifically binds to human bladder transitional cell carcinoma (TCC) cell lines and to cancer cells obtained from five different clinical patient specimens *in vitro *and *in vivo *[1]. Currently, we are conducting the preclinical studies to determine the diagnostic and targeted therapeutic potential of PLZ4. One major problem we have encountered is that the mouse models commonly used for preclinical studies are neither physiologically nor physically applicable to human bladder cancer. In contrast, dogs with naturally occurring bladder TCC can serve as a spontaneous, outbred, immune-competent, large animal model for TCC in the human [2,3]. Bladder tumors in dogs possess similar histopathological appearance, molecular features, biological behavior, and response to chemotherapy as do bladder TCC in humans [4,5]. Therefore, we determined whether PLZ4 could bind to canine bladder cancer. If so, this peptide can potentially be developed for the diagnostic and therapeutic purposes in the management of canine bladder cancer, since bladder TCC is the most common urinary cancer in dogs (87% of all cases) [6].

## Findings

**Figure 1:**
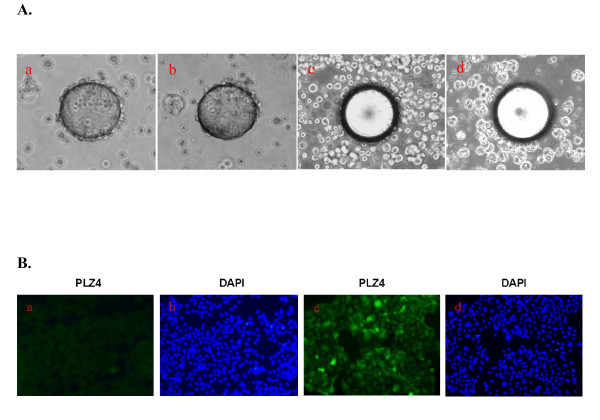
**PLZ4 binds to Canine TCC cells**. A. whole cell binding assay to determine cell binding specificity of PLZ4. Cells were resuspended at 10^6 ^cells/ml and incubated with beads coated with PLZ4. If PLZ4 binds to cells in solution, bead surface would be covered with cells and exhibit a rosette pattern under the microscopy examination. This experiment was repeated 3 times for cell lines. The cell binding assay of normal canine bladder urothelial cells was repeated on 3 different dogs. **a**. 5637 human bladder cancer cell line; **b**. K9TCC-PU cell line; **c**. normal canine urothelial cells; **d**. urothelial cells from a bladder with chronic cystitis. The average diameter of the beads is 90 μm. B. Affinitofluorescence of PLZ4 peptide toward Canine TCC cell lines. Affinitofluorescence staining was performed with all five canine TCC cell lines and normal canine bladder urothelial cells of dogs euthanized for non-bladder diseases. Only fluorescence staining to normal urothelial cells (a) and K9TCC-PU cells (c) was shown. b and d showed the corresponding DAPI nuclear staining of a and c, respectively.

To determine the binding of PLZ4 to canine invasive TCC cell lines, a whole cell binding assay was performed (Additional File 1). We first synthesized PLZ4 on TentaGel S NH2 resin beads (Rapp Polymere Gmbh, Germany) [1,7], and incubated the PLZ4-coated beads with 10^6 ^cells/ml single-cell suspensions of five different canine invasive TCC cell lines including K9TCC-PU, K9TCC-PU-AxA, K9TCC-PU-In, K9TCC-PU-AxC, and K9TCC-PU-Nk (kindly provided by Deborah Knapp at Purdue University, West Lafayette, IN, USA) [3]. Human bladder cancer cell line 5637 cells served as the positive control. Over 95% of the bead surface was covered with 5637 and K9TCC cells (Figure 1A: a and 1A: b, respectively). In contrast, there was no cell binding and a smooth bead surface was observed when the beads were incubated with the normal canine bladder urothelial cells (Figure 1A: c), or bladder cells from a dog with chronic cystitis (Figure 1A: d).

To further evaluate the binding of PLZ4 to canine TCC cell lines, we conducted an affinitofluorescence assay. PLZ4 was synthesized and covalently conjugated to biotin. Canine TCC cell lines were cultured on chamber slides. Normal urothelial cells from dogs euthanized for non-bladder diseases were isolated and then made into a single-cell suspension before cytospin slide preparations were performed. Slides were fixed with acetone for 2 min before blocking. Cells were incubated with 1 μM of PLZ4-biotin for 1 hour at 4°C, then with SA-Alexa Flour^® ^488 conjugate (Invitrogen, Carlsbad, CA, USA) at 1:1000 dilution per manufacturer's protocol. After washing, the slides were mounted with DAPI-containing medium for nuclear staining (blue), and observed under inverted fluorescence microscope (200 X). All five canine TCC cell lines showed diffuse cell membrane staining (Figure 1 B: c. fluorescence staining of K9TCC-PU), while no significant staining was observed with normal canine urothelial cells (Figure 1B: a).

**Figure 2:**
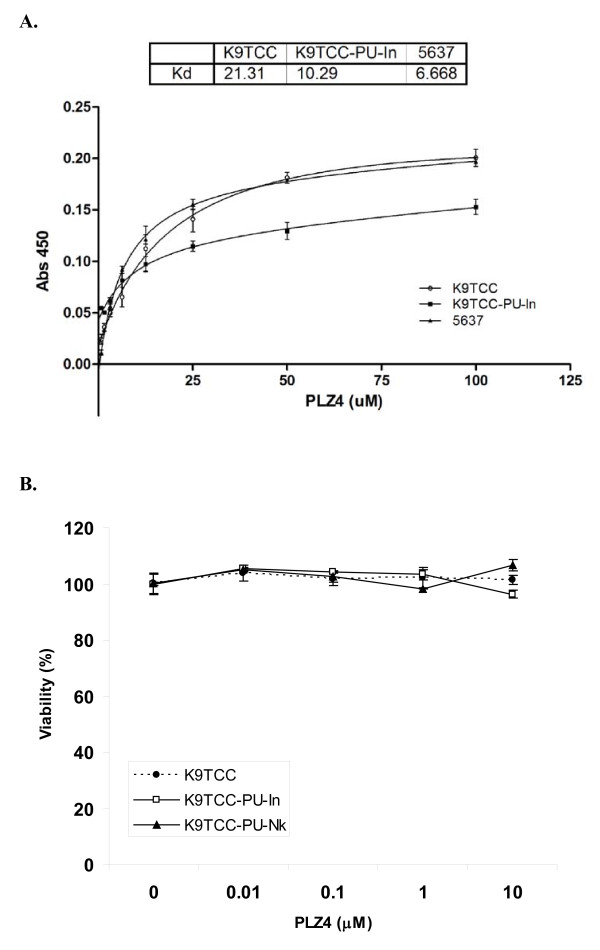
**Binding Affinity and biological effects of PLZ4 against canine TCC cell lines**. A. Binding affinity of PLZ4 against K9TCC-PU and K9TCC-PU-In. Twenty thousands cells of K9TCC-PU and K9TCC-PU-In were seeded in 96 well plates. After culture for 24 hours, cells were fixed and incubated with different concentrations of PLZ4-biotin for 1.5 hours followed by SA-HRP for another 1 hour. Cells treated with SA-HRP alone served as background control. The color was developed using TMB substrate and read by ELISA readers. Three independent experiments conducted in triplicate were performed. The mean values of the 3 experiments are shown. B. Biological effects of PLZ4 on canine TCC cell lines. Ten thousand cells of K9TCC-PU-In or K9TCC-PU were seeded in the 96-well plates and treated with increasing concentrations of PLZ4 or PBS for 2 days. The cell proliferation assay was assessed by the WST-8 assay per manufacturer's protocol. Cells treated with PBS were used as 100% control. Each experiment was performed three times in triplicate. Mean values at each concentration are presented.

To further quantify the binding affinity, K9TCC-PU and K9TCC-PU-In cells were seeded in 96-well plates, fixed with acetone, and incubated with increasing concentrations of PLZ4-biotin followed by SA-HRP. As shown in Figure 2A, PLZ4 exhibited a dose-dependent binding against canine TCC cell lines. The Kd_50 _values of PLZ4 for K9TCC-PU and K9TCC-PU-In (the concentration of PLZ4 to saturate 50% of cell surface receptor) were 21.3 and 10.3 μM, respectively, while the Kd_50 _value for human 5637 cells was 6.67 μM.

Ligand binding to cell surface molecules may initiate cell signaling and exert biological effects on cells. Here, we determined the effect of PLZ4 on cell viability and proliferation, as these PLZ4-mediated effects may have potential clinical applications. K9TCC, K9TCC-PU-In and K9TCC-PU-Nk cells were seeded in 96-well plates and incubated without or with various concentrations of PLZ4 for 48 hours. The WST-8 cell proliferation assay was performed per manufacturer's protocol (Cayman Chemical, Ann Arbor, MI, USA). There were no significant changes in cell proliferation/viability in these three cell lines cultured with different concentrations of PLZ4 when compared to the control cells treated with PBS (Figure 2B).

**Figure 3:**
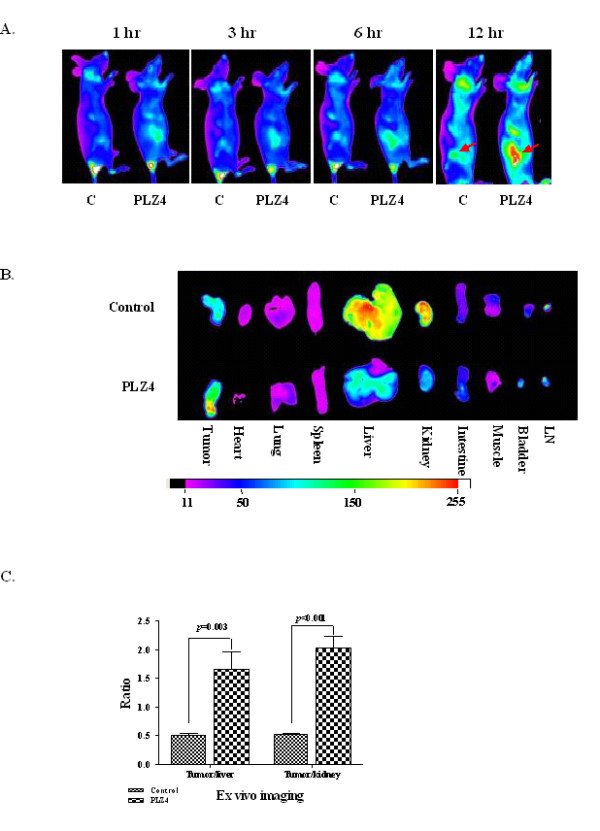
**Homing of PLZ4 to mouse xenograft of canine bladder cancers**. Nude mice with the xenografts from TCC-PU-In at 0.5-0.8 cm in diameter were randomly selected to be injected with 100 μl (6 nmol) of pre-incubated PLZ4-biotin-SA-Cy5.5 complex or SA-Cy5.5 dye as the control. Total body imaging was performed at 0, 1, 3, 6, and 12 hours after injection. All experiments were conducted in compliance with institutional guidelines and according to the protocol (No. 12988) approved by the Institutional Animal Care and Use Committee of the University of California at Davis. A. *In vivo *imaging of canine K9TCC-PU-In xenografts with PLZ4. I*n vivo *near-infrared fluorescence images were taken at different time points after injection. C: the control mouse that received SA-Cy5.5. PLZ4: the mouse that received PLZ4-Cy5.5. Red arrows point to tumor xenografts. B. E*x vivo *imaging of organs and tumor xenografts for fluorescence intensity. A color bar with the fluorescence intensity in arbitrary units is shown at the bottom. C. *Ex vivo *quantitative analysis of fluorescence uptake in tumor xenografts. The fluorescence intensity of tumor xenografts was normalized to that of the liver and kidney of the same mouse (the normalized value of liver and kidney is defined as 1.0).

Next, we determined the tumor-specific homing/targeting property and *in vivo *biodistribution/binding specificity of PLZ4 in a mouse model with canine TCC xenografts. The near-infrared fluorescent dye Cy5.5 was used for *in vivo *and *ex vivo *imaging. PLZ4-biotin-SA-Cy5.5 (PLZ4-Cy5.5) complex or SA-Cy5.5 dye (control), 100 μl (6 nmol) for each mouse, was injected via tail vein into mice carrying TCC-PU-In xenografts at 0.5-0.8 cm in diameter. Substantial accumulation of signals was observed at the tumor site in the mice injected with PLZ4-Cy5.5 complex in a time-dependent manner with a maximum signal observed at 12 hours (Figure 3A). In contrast, negligible fluorescence uptake of Cy5.5 dye by tumors was detected in the control mice receiving SA-Cy5.5. To determine if there was any non-specific uptake of the dye by other vital organs, mice were euthanized at 12 hours after injection, vital organs and cancer xenografts were removed for *ex vivo *imaging (Figure 3B). Both liver and kidney demonstrated considerable signals even in the control mice that received SA-Cy5.5, suggesting the non-specific uptake. Compared with the tumor xenografts from the control mice treated with SA-Cy5.5, xenografts from the mice that received PLZ4-Cy5.5 accumulated significantly higher fluorescence signals after normalization with the fluorescence signal in liver (3.2 times, *p *= 0.003) and kidney (3.8 times, *p *< 0.001) (Figure 3C). No significant fluorescence uptake was observed in other organs including bladder. Collectively, these data demonstrated that PLZ4 exhibited excellent homing property toward TCC xenografts *in vivo*.

## Discussion

This is the first study showing that a human bladder cancer-specific peptide can also target canine bladder TCC cells. During drug development, immunocompromised mice with tumor xenografts are most often used for preclinical studies. Physiologically, mouse xenograft models are radically different from naturally occurring cancer in human patients. The most commonly used xenograft model is subcutaneous xenografts while most human cancers, even at the very late stage, rarely metastasize to the subcutaneous space. Furthermore, because of the rapid tumor formation (weeks) in xenograft models, the local vasculature formation and permeability can be dramatically different from that of naturally occurring cancer that usually take months to years to develop. Here we showed that PLZ4 can bind to canine bladder cancer cells both *in vitro *and *in vivo *(Figures 1 and 3). Consistent with our previous finding that PLZ4 could bind to primary bladder cancer cells from five different human patients but not to urine cells collected from four cancer-free patients actively receiving Bacillus Calmette-Guérin treatment [1], PLZ4 could bind to cancer cells from one canine bladder TCC clinical specimen, but not to a bladder lymphoid hyperplasia specimen from another dog patient (data not shown), or a bladder with chronic cystitis (Figure 1d). Our findings suggest that most of the preclinical studies of PLZ4 can be performed and validated in dogs with naturally occurring bladder cancer. Furthermore, the US Food and Drug Administration requires that the pharmacology and toxicology studies in two species of mammalian animals (usually in rats and dogs) be performed before the first-in-human study can be conducted.

One major application of PLZ4 is local visualization of bladder cancer during transurethral resection of bladder cancer (TURBT). It is clinically relevant since incomplete resection is seen in up to one third of cases after TURBT that contributes to the high recurrence of bladder cancer after therapy [8]. Fluorescence cystoscopy with 5-Aminolevulinic acid (ALA) has been used for this purpose [9]. But nonspecific uptake of ALA by non-cancer urothelial cells, especially in inflamed bladder, precludes its wide clinical application. PLZ4 binds to canine bladder cancer cells, but not to normal urothelial cells or cells from a bladder with chronic cystitis (Figure 1). This suggests that fluorescence cystoscopy with PLZ4 conjugated to a fluorescent dye can be an excellent candidate for this application, and that dogs with naturally occurring bladder cancer can be an outstanding model. The Kd50 of PLZ4 at 10.29 and 21.31 μM can be easily achievable with local intravesical instillation.

Our *in vivo *targeting and biodistribution study showed that PLZ4 specifically concentrated at the tumor sites, but not to other sites including bladder (Figure 3). Therefore, PLZ4 has the potential to be developed for imaging detection of bladder cancer by conjugated to imaging agents, such as iron oxide for MRI (magnetic resonance imaging) and radioisotope for PET/SPECT (positron emission tomography/single photon emission computed tomography). Both MRI and PET scans have been used for the diagnosis of canine malignancies. Another potential application is targeted therapy by conjugating PLZ4 to toxin or chemotherapeutic drugs. In our *in vivo *studies (Figure 3), only 6 nmol (equivalent to 17 mg of PLZ4 in a 75-Kg patient) of PLZ4-Cy5.5 was used, and little non-specific uptake was observed. This is consistent with our previous findings that PLZ4 did not bind to any of the confounding cells that possibly exist inside the bladder such as normal urothelial cells, whole blood, peripheral blood mononuclear cells, fibroblasts, and vascular endothelial cells. This specific binding is critical for *in vivo *targeting with PLZ4 conjugates.

In summary, the human bladder cancer-specific peptide PLZ4 can bind to canine bladder cancer cells, suggesting that the preclinical studies of PLZ4 can be performed in dogs with naturally occurring bladder TCC.

## List of abbreviations

**TCC**: Transitional cell carcinoma; **TURBT**: transurethral resection of bladder cancer; **SA**: streptavidin; **SI**: Supplement information; **ALA**: 5-Aminolevulinic acid; **MRI**: magnetic resonance imaging; **PET/SPECT**: positron emission tomography/single photon emission computed tomography; **PLZ4**: the name of the bladder cancer-specific ligand.

## Competing interests

Bin Li has signed an option with UC Davis to license the patent of PLZ4 for commercial development. We have no other financial competing interests to disclose.

## Authors' contributions

TL: carried out the affinitofluorescence, binding affinity, and biological effects of PLZ4 on canine TCC cells, data acquisition and analysis, and manuscript preparation. HZ: developed PLZ4 ligands and contributed to the conception and design of the experiments. SW: conducted the *in vivo *and *ex vivo *imaging and biodistribution studies, and analyzed the data. LX and BL: Initiated this project, conducted the whole bead cell binding and first affinitofluorescence staining. COR: collected canine tissue and helped in the determination the binding of PLZ4 to normal canine urothelial cells and cells with chronic cystitis. RDVW: design and supervision of the overall project, data analysis and manuscript preparation. CP: principal investigator of this project, contributed to conception and design of the project, manuscript preparation and final approval. All authors read and approved the final manuscript.

## Author's information

TL, HZ, SW, LX and BL are current or former trainees of Dr. Pan's lab. COR is a Veterinary Medical oncologist and a Continuing Lecturer. RDVW is the director of the UC Davis Cancer Center and Professor of Urology. CP received 4 years of residency training in Urology in China, finished residency and fellowship training and is board-certified in Internal Medicine, Hematology and Oncology in US. He also had PhD in Microbiology & Immunology. Currently, he is a physician-scientist and an Assistant Professor at the Division of Hematology/Oncology, Department of Internal Medicine, UC Davis Medical Center.

## Supplementary Material

Additional file 1 **Materials and Methods**. A detail description of the materials and methods for this publication. Click here for file
