# Supplementary material for: Targeting canine bladder transitional cell carcinoma with a human bladder cancer-specific ligand
Source: Mol Cancer. 2011 Jan 27;10:9. doi: 10.1186/1476-4598-10-9 (PMC3040722; doi:10.1186/1476-4598-10-9)
Supplement: Additional file 1 — Materials and Methods. A detail description of the materials and methods for this publication. [file 1476-4598-10-9-S1.DOC]

**Cancer-Specific peptide for canine TCC-Supplemental Information**

**Materials and Methods:**

**Synthesis of peptide**

The cyclic peptide PLZ4-biotin (the amino acid sequence : cQDGRMGFc) was obtained using solid-phase synthesis as previously described [1, 2].

**Cell lines and culture condition**

Five invasive canine transitional cell carcinoma cell lines including K9TCC, K9TCC-PU-Pu, K9TCC-PU-In, K9TCC-PU-AxC, and K9TCC-PU-NK were kindly provided by Deborah Knapp DVM, PhD at Purdue University [3]. These cell lines were maintained in the DMEM/F12(1:1) (Invitrogen, Carlsbad, CA) medium supplied with 10% FBS, Penicillin-streptomycin, non-essential amino acid, and sodium pyruvate. The human bladder cancer line, 5637 was purchased from ATCC (Manassas, VA), and cultured in the RPMI1640 supplied with 10% FBS, penicillin-streptomycin. Cells were passaged every 2-3 days. Normal canine urothelial cells were collected from three different dogs euthanized for non-bladder-related diseases and this process was authorized by the owners. A small piece of bladder was removed and a thin layer of bladder urothelial cells were scrubbed by surgical blade. After washing with 3 times of PBS, cells and smaller pieces of superficial tissues were incubated with 1.5 ml of Accutase® (Invitrogen, Carlsbad, CA) at 37^o^C water bath for 40 minutes with short vortex periodically. Cells were then washed with complete medium three times and used in the whole cell binding test. The viability of large urothelial/epithelial cells was over 95% as evaluated by Trypan Blue staining. Slight blood contamination was noted.

**Whole cell binding assay**

Five canine TCC cell lines were washed with PBS, detached from tissue culture dishes with a 5 minute digestion with trypsin, and then made into single cell suspension by gentle pipetting. After washing three times with PBS, cancer cells or the normal urothelial cells were re-suspended into 10^6^/ml of warm complete medium in 60 mm culture dishes. PLZ4 was synthesized on solid phase TentaGel S NH2 resin beads (Rapp Polymere Gmbh, Germany) [1, 2]. Up to 10^13^ copies of PLZ4 could be synthesized on a single 90 µm diameter bead. Ten μl of PLZ4 beads (7,500 beads) were thoroughly washed once with water and twice with PBS, and then added to the single-cell suspension. Incubation for 60 minutes with gentle shake at 60 rpm at 37^o^C was allowed. The results were observed under inverted microscope. This experiment was repeated 3 times on each canine cell line, and 3 times on canine normal urothelial cells from three different dogs, respectively.

**Affinitofluorescence Essay**

Five canine cancer cell lines were cultured in 8-chamber slides (BD bioscience, San Jose, CA ) overnight in 500 μl of complete culture medium. To obtain slides with normal canine bladder urothelial cells, bladder was obtained from dogs euthanized for non-bladder diseases. Urothelium was scraped from the bladder and digested with accutase per manufacturer’s protocol to make a single-cell suspension. Normal urothelial cells were then cyto-spun to slides. Slides were air dried and fixed with acetone for 2 minutes. After washing with PBS, slides were blocked in 1% FBS/0.5% Tween20/PBS (wash buffer) for 20 minutes followed by 1 μM of PLZ4 for 1 hour at 4^o^C. After incubation with PLZ4, the slides were washed with wash buffer 3 times and incubated with 0.5 µg/ml of Streptavidin (SA)-Alexa Fluor® 488 Conjugate (Invitrogen/Molecular Probe, Carlsbad, CA) for another 1 hour at 4 ^o^C. Cells incubated with only SA-Alexa Fluor® 488 served as a control for non-specific binding. Prior to examination under the fluorescent microscope, the slides were mounted with DAPI (Invitrogen/Molecular Probe, Carlsbad, CA) for nuclear staining and covered. These experiments were repeated 3 times.

**Cell viability assay**

Ten thousand cells of canine TCC cell lines K9TCC-PU, K9TCC-PU-In and K9TCC-PU-Nk, were seeded in 100 μl complete culture medium in the 96 well plates and allowed to attach overnight. Cells were treated with PBS or various concentrations of PLZ4-biotin conjugate at indicated concentrations. After 2 days of continuous incubation, 10 μl of WST-8 (Cayman, Ann Arbor, MI) working solution was added to the plates and incubation continued for another 1.5-2 hours at 37^o^C. The wells with only medium and 10 μl of WST-8 solution served as background control, and cells treated with PBS served as the 100% control. The plates were read by the ELISA reader (Invitrogen, Carlsbad, CA). These experiments were performed three times in triplicate.

***In vivo* and *ex vivo* mouse imaging**

To investigate the potential application of PLZ4 for *in vivo* imaging, we conducted an experiment with a mouse xenograft model using the near-infrared fluorescence (NIRF) optical imaging system. All the animal experiments followed the institutional guideline and the animal protocol (No. 12988) was approved by the Animal Use and Care Administrative Advisory Committee of the University of California-Davis. Ten eight-week-old athymic nude mice were purchased from Harlan Laboratories (Indianapolis, IN). TCC-PU-In cells (10^7^ cells) were washed 3 times in PBS and mixed with Matrigel® (BD Biosciences, San Jose, CA) at a 1:1 ratio, and injected subcutaneously on the right lower flank of each mouse. Mice were monitored once weekly for tumor xenograft formation. When the xenografts measured 0.5-1.0 cm, the mice were considered ready for imaging studies. One streptavidin (SA) molecule can bind up to 4 molecules of biotin. To ensure at least one PLZ4-biotin molecule bound to SA-Cy5.5, we mixed PLZ4-biotin with SA-Cy5.5 (NANOCS, New York, NY) at molar ratio of 5:1 for 1 h at 4^o^C. We confirmed the fluorescence labeling by *in vitro* cell-binding assays. Mice were anesthetized by intraperitoneal injection of pentobarbital sodium and either 100 μl of SA-Cy5.5 or 100 μl of PLZ4-biotin-SA-Cy5.5 complex (6 micromole) was injected via the tail vain. Whole body imaging was collected at 0, 1, 3, and 12 hours using a Kodak multimodal-imaging system IS2000MM (Kodak, Rochester, NY) with an excitation filter at 625 nm and an emission filter at 700 nm. Mice were sacrificed after the last time point, and the major organs and tumors were removed for *ex vivo* imaging. All images were further analyzed by the software, “Imaging station IS2000MM”. The experiment was repeated 3 times.

**Statistics**

The experiments were repeated in duplicate or triplicate. The data were presented as mean +/- SD. The student *t*-test was conducted and the *p*<0.05 was considered as significant. For determination of tumor uptake, we calculated mean fluorescence intensities of the tumor area and of the normal tissue area by means of the region-of-interest function using Kodak 1D Image Analysis Software (Kodak), then plotted a pseudocolored scale based the semiquantitative information from NIRF images by integrating fluorescence intensities from equal areas within tumor and normal tissue regions

**References:**

1. Zhang H, Aina OH, Lam KS, de Vere White R, Evans C, Henderson P, Lara PN, Wang X, Bassuk JA, Pan CX: **Identification of a bladder cancer-specific ligand using a combinatorial chemistry approach.** *Urol Oncol* 2011.

2. Peng L, Liu R, Marik J, Wang X, Takada Y, Lam KS: **Combinatorial chemistry identifies high-affinity peptidomimetics against alpha4beta1 integrin for in vivo tumor imaging.** *Nat Chem Biol* 2006, **2:**381-389.

3. Dhawan D, Ramos-Vara JA, Stewart JC, Zheng R, Knapp DW: **Canine invasive transitional cell carcinoma cell lines: in vitro tools to complement a relevant animal model of invasive urinary bladder cancer.** *Urol Oncol* 2009, **27:**284-292.
